# Effective processing pipeline PACE 2.0 for enhancing chest x-ray contrast and diagnostic interpretability

**DOI:** 10.1038/s41598-023-49534-y

**Published:** 2023-12-18

**Authors:** Giulio Siracusano, Aurelio La Corte, Annamaria Giuseppina Nucera, Michele Gaeta, Massimo Chiappini, Giovanni Finocchio

**Affiliations:** 1 Department of Electric, Electronic and Computer Engineering, University of Catania, Viale Andrea Doria 6, 95125 Catania, Italy; 2 Unit of Radiology, Department of Advanced Diagnostic-Therapeutic Technologies, “Bianchi-Melacrino-Morelli” Hospital, Reggio Calabria, Via Giuseppe Melacrino, 21, 89124 Reggio Calabria, Italy; 3 Department of Biomedical Sciences, Dental and of Morphological and Functional Images, University of Messina, Via Consolare Valeria 1, 98125 Messina, Italy; 4 Istituto Nazionale di Geofisica e Vulcanologia (INGV), Via di Vigna Murata 605, 00143 Rome, Italy; 5 Maris Scarl, Via Vigna Murata 606, 00143 Rome, Italy; 6 Department of Mathematical and Computer Sciences, Physical Sciences and Earth Sciences, University of Messina, V.le F. Stagno D’Alcontres 31, 98166 Messina, Italy

**Keywords:** Medical imaging, Radiography

## Abstract

Preprocessing is an essential task for the correct analysis of digital medical images. In particular, X-ray imaging might contain artifacts, low contrast, diffractions or intensity inhomogeneities. Recently, we have developed a procedure named PACE that is able to improve chest X-ray (CXR) images including the enforcement of clinical evaluation of pneumonia originated by COVID-19. At the clinical benchmark state of this tool, there have been found some peculiar conditions causing a reduction of details over large bright regions (as in ground-glass opacities and in pleural effusions in bedridden patients) and resulting in oversaturated areas. Here, we have significantly improved the overall performance of the original approach including the results in those specific cases by developing PACE2.0. It combines 2D image decomposition, non-local means denoising, gamma correction, and recursive algorithms to improve image quality. The tool has been evaluated using three metrics: contrast improvement index, information entropy, and effective measure of enhancement, resulting in an average increase of 35% in CII, 7.5% in ENT, 95.6% in EME and 13% in BRISQUE against original radiographies. Additionally, the enhanced images were fed to a pre-trained DenseNet-121 model for transfer learning, resulting in an increase in classification accuracy from 80 to 94% and recall from 89 to 97%, respectively. These improvements led to a potential enhancement of the interpretability of lesion detection in CXRs. PACE2.0 has the potential to become a valuable tool for clinical decision support and could help healthcare professionals detect pneumonia more accurately.

## Introduction

Chest X-Ray (CXR) is a low-cost, widely available, practical screening technique in the diagnosis of multiple pulmonary diseases, like pneumonia, which is among the top infectious causes of death for children around the world^1^. Recently, CXRs have been massively used for detecting and monitoring COVID-19 being less harmful to the human body compared to CT, where effective radiation dose is about 50-fold higher than the average effective dose for CXR (posteroanterior and lateral projection)^2,3^. Nonetheless, despite the ease of acquisition, the interpretation of a chest X-ray can be challenging^4^.

Recently, it has been demonstrated that image enhancement advances the interpretability of medical images, both improving feature extraction for deep learning approaches and creating a better visual representation for human viewers^5^. The improvement is not only subjective but can be measured objectively via key performance indicators^6^. However, the main challenge is to preserve the original information avoiding to introduce artifacts during the image enhancement process. Various techniques have been considered in the literature for this purpose, histogram transformations^7–9^, de-noising algorithms^10,11^, filtering^12^, decomposition^13–15^, and interpolation^16–18^ to cite a few. Some methods are effective in compensating low contrast^19,20^ or luminance inhomogeneities^21^, foreign objects detection^22^, or enhancing the geometric features such as edges, corners, and ridges of the medical images^23–27^. Over the past years, numerous local image enhancement algorithms have been introduced with the dual objective of enhancing image quality and optimizing the performance of machine learning (ML) models^28–30^. Salem et al.^31^ investigated the use of different histogram equalization techniques, which can help in image enhancement. Alavijeh and coworkers in Ref.^32^ further enhanced this approach by combining contrast-limited adaptive histogram equalization with morphological top-hat and bottom-hat filtering. This resulted in a notable improvement of image contrast while retaining critical information from the chest tissue.

Chen et al.^33^ described a method for enhancing chest images via bone suppression. Firstly, they applied a region of interest-based look-up table to standardize CXRs. Then, an artificial neural network was trained with regular CXRs and the corresponding images without bones using dual-energy subtraction (DES). Although it was applied to a limited number of patients, results demonstrated how bone structures were suppressed while simultaneously preserving subtle pathological changes. Mohammad et al.^34^ introduced bi-and multi-histogram methods designed to enhance image contrast while preserving brightness and a natural appearance of the images. This latter technique has been useful in many applications that require image enhancement^35–37^. In a recent study, Nefoussi et al.^38^ showed how to enhance CXR images by combining different image pre-processing techniques such as histogram equalization, CLAHE, and unsharp mask.

Several other popular histogram techniques were also explored for CXR images to investigate whether they can help ML models in various tasks such as image enhancement^9,13^, classification^39,40^, or anomaly detection^41^. From literature, it clearly emerges the importance and the scientific motivations of why effective image processing is necessary.

Here, we will focus on image enhancement tools to help clinicians to make an accurate diagnosis of diseases by using CXRs^5,42,43^. We wish to emphasize that although easy to generate, CXR images can be affected by a variety of detrimental factors including: low-quality issues induced by environment difficulties or phenomena^8,44,45^, non-collaborative or severely ill patients^46,47^ causing many different artifacts like debris or gain calibration flaws^48^, problems during acquisition, inhomogeneities of luminance distribution^49^. Our previous post-processing tool presented in Ref.^13^, named Pipeline for Advanced Contrast Enhancement (PACE) was developed for automatically enhancing CXR images enabling better detection of lung lesions. An extensive use for benchmarking this tool by two Italian hospitals one in Messina and one in Reggio Calabria has shown some critical conditions where the algorithm underperforms. In particular, it triggers an artifact that is a reduction of details over large bright regions (as in pleural effusions) originating in oversaturated areas. Here, we have developed a new version of the algorithm (PACE2.0) to overcome these drawbacks that can impact diagnostic effectiveness and to further improve the interpretability of information contained in the radiographic image by removing certain artifacts.

The progress of the method has been achieved via a combination of techniques that are used to level up the visual appearance of an image. A 2D image decomposition technique is combined with non-local means denoising to enable independent filtering of different components of the image. Then, a gamma correction of the image is applied to make details more visible. Subsequently, image contrast is enhanced using CLAHE and a recursive procedure solves a complex multi-objective optimization problem. The overall performance of PACE2.0 has been evaluated in terms of well-established metrics such as image entropy (ENT), contrast improvement index (CII), Effective Measure of Enhancement (EME) and Blind/Referenceless Image Spatial Quality Evaluator (BRISQUE) using both proprietary and public datasets. A theoretical description of most of these parameters is also provided in Ref.^13^. We also show how PACE2.0 can be used as an effective tool to boost medical image classification performance based on Deep Learning (DL) models. We report an enhancement from 80 to 94% for the classification accuracy and from 89 to 97% of the recall against unprocessed CXRs which has been achieved here by using the state-of-the-art DL architecture DenseNet-121^50^ benchmarked on a publicly available database (https://www.kaggle.com/c/rsna-pneumonia-detection-challenge^51^).

## PACE2.0 algorithm architecture

**Figure 1:**
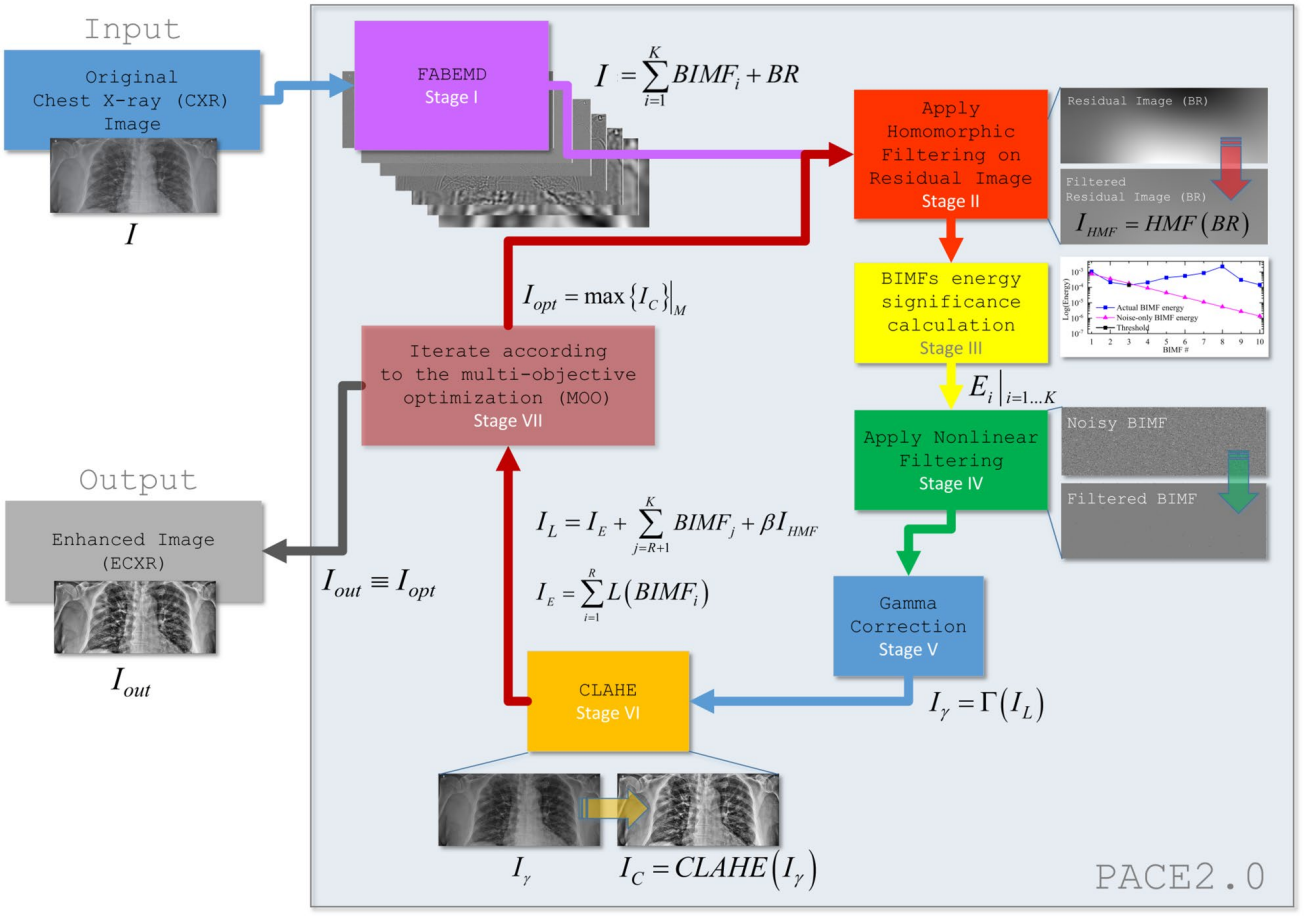
A block diagram of PACE2.0 method as developed in this work. The method is designed to convert input chest X-ray (CXR) images into enhanced CXR (ECXR) images through a series of different steps. (1) FABEMD generates the bi-dimensional intrinsic mode functions and residual image, (2) the HMF stage is used to filter the BR while BIMF energy significance is calculated (3). In (4) Nonlinear Filtering is performed on the least significant BIMFs and the image is reconstructed (by combining BIMFs and BR). (5) Gamma correction is applied on the reconstructed image *I*_*L*_, to generate *I*_*γ*_. (6) Then, CLAHE is executed on the GC image *I*_*γ*_ to improve the overall contrast and compute *I*_*C*_. Now, steps (2–6) are repeated iteratively until the best result is given under MOO criteria (7), *I*_*opt*_. Then, the best result is chosen, and the enhanced image is generated and provided as output *I*_*out*_.

A complete sketch of the block diagram of PACE2.0 is shown in Fig. 1.

1. The first step is the decomposition of the CXR image $I$ into a finite number of *K* bi-dimensional intrinsic mode functions BIMF_*i*_ (*i* = 1… *K*) and a bi-dimensional residual image (BR) by using the Fast and Adaptive Bi-dimensional Empirical Mode Decomposition (FABEMD)^52^ to have the following relationship: $I = \sum\limits_{i = 1}^{K} {BMF_{i} + BR}$. We set *K* = 10.
2. The residual image, *BR*, is then processed with a Homomorphic Filter (HMF) where the kernel function is a High-Frequency Emphasis Filter (HEF) to obtain *I*_*HMF* _= *HMF* (*BR*).
3. The energy $E_{i} \left| {_{i = 1,...K} } \right.$ of the BIMFs is calculated using the method described in Ref.^56^. Here, $E_{i}$ is the energy associated to the *i*-th BIMF component of the input image.
4. The *R* lowest energy components (where $R < K$ and represents the number of BIMFs whose energy is closer to the energy of noise-only signals) are denoised via nonlinear filtering, such that the *i*-th filtered BIMF can be written as $L\left( {BIMF_{i} } \right)$. Using this formulation we can represent the sum of denoised BIMFs as $I_{E} = \sum\limits_{i = 1}^{R} {L\left( {BIMF_{i} } \right)} + \sum\limits_{j = R + 1}^{K} {BIMF_{j} }$. Subsequently, the noise-reduced image $I_{L}$ is reconstructed by combining $I_{E}$ defined as the sum of the first *R* denoised BIMFs and $\left( {K - R} \right)$ original BIMFs, and the filtered BR, $I_{L} = I_{E} + \beta I_{HMF}$. The control parameter $\beta$ is in the range [0,1] and its role will be explained in Section II.
5. The Gamma Correction (GC) is then applied on the reconstructed image $I_{L}$ to mitigate over-exposed as well as underexposed areas, *I*_*γ* _= Γ(*I*_*L*_).
6. The image is processed by using the CLAHE algorithm, such that $I_{C} = CLAHE\left( {I_{\gamma } } \right)$. $I_{C}$ is then used to extract the metrics ENT, CII and EME as discussed in Ref.^13^.
7. The procedure described within the steps from (2) to (6) is iteratively repeated using a Multi-Objective Optimization (MOO) algorithm based on the variability range of the defined parameters. This is performed in order to obtain the highest possible score combining the considered set of metrics (i.e. ENT, CII and EME). The optimum result of (7) generates the enhanced CXR (ECXR) as computed with PACE2.0. Table 1 summarizes the role of the main blocks which were briefly introduced above and will be described in detail in the following pages.

**Table 1:** Outline of PACE2.0 with the description of each computational step, the considered technique, features, and intermediate output.

Step	Method	Description	Features	PACE—vs.—PACE2.0	Output
1	FABEMD^52^	Decomposes an image into multiple hierarchical components known as Bi-dimensional Intrinsic Mode Functions (BIMFs) and bi-dimensional residue	This method offers a means to achieve rapid results with reduced computational effort, while also ensuring a more precise estimation of the BIMFs. In addition, FABEMD is more adaptive to handle large size input than the traditional BEMD. Furthermore, the FABEMD is less prone to boundary effects and overshoot-undershoot problems^53^	Used in both PACE and PACE2.0	An input image is divided into a set of independent components (BIMFs) and a bi-dimensional residue
2	HMF^54^	Homomorphic filtering is a popular and effective image enhancement technique that can achieve two important objectives in a single step. Specifically, it simultaneously normalizes the brightness and enhances the contrast of an image^55^	Here homomorphic filtering is used to remove luminance inhomogeneities on the residual image	Used in both PACE and PACE2.0	The residual image is filtered to correct luminance inhomogeneities
3	BIMF Energy calculation^56^	The energy of BIMFs is calculated according to the proposed method	It is possible to classify which components are mostly made of noise and which contain relevant information	PACE2.0	Energies of each BIMF. Relevance of BIMFs is calculated
4	Nonlinear Filtering^57^	A non-linear digital filtering technique is used to remove impulsive noise from the signals while preserving the edges	Here it is used to remove the impulsive noise from specific BIMFs. The image is then reconstructed by recombining the different components after selective processing	PACE2.0	Reconstructed image with denoised BIMFs (mitigated impulsive noise) and filtered residual
5	Gamma Correction^58^	Gamma Correction is used to appropriately adjust the brightness of the image based on the image information	Here it is used to prepare the image for contrast enhancement and reduce the over-exposed areas which might be the cause of adverse effects (i.e. artifacts)	PACE2.0	Gamma corrected image
6	CLAHE^59^	CLAHE performs contrast amplification using a limiting procedure that is applied for each neighboring pixel which then forms a transformation function to reduce the noise problem	CLAHE is computed in conjunction with GC to improve luminance and contrast	Used in both PACE and PACE2.0	Reconstructed image with enhanced contrast
7	MOO^60,61^	Multi-objective optimization	MOO is applied to generate the best results according to the considered performance metrics. A total of 120 combinations of parameters were considered for PACE, whereas 256 permutations are considered for PACE2.0	Used in PACE. Enhanced in PACE2.0	Produces the best results by evaluating a combination of proposed metrics

### Fast and adaptive bi-dimensional empirical mode decomposition—FABEMD

FABEMD^62^ has been proposed in the literature as a computationally-efficient version of the Bi-dimensional Empirical Mode Decomposition (BEMD)^63^. The technique is already part of the original PACE algorithm and it has been described in detail in Ref.^13^. The purpose of FABEMD is to decompose an input image into a finite number of characteristic components (called Bi-dimensional Intrinsic Mode Functions or BIMFs) each carrying a specific subset of details plus a BR image which contains the luminance background information. These BIMFs are sorted based on their frequency content and their contribution to the input image.

### Homomorphic Filtering—HMF

Homomorphic filtering is a widely used signal and image processing technique, employs a nonlinear mapping to a different domain where linear filter techniques are applied, and then reverts to the original domain through another mapping. The technique is already part of the original PACE algorithm and it has been described in detail in Ref.^13^. HMF is applied to the BR of the input image. Differently from the original implementation, PACE2.0 introduces a specific control parameter $\beta = [0,1]$ which evaluates whether to include or exclude the filtered BR from further processing steps when his absence or presence might prevent artifacts generation and lead to better overall performance. This parameter comes into place when reconstructing the image before it is processed using CLAHE.

### Energy calculation of the BIMFs

**Figure 2:**
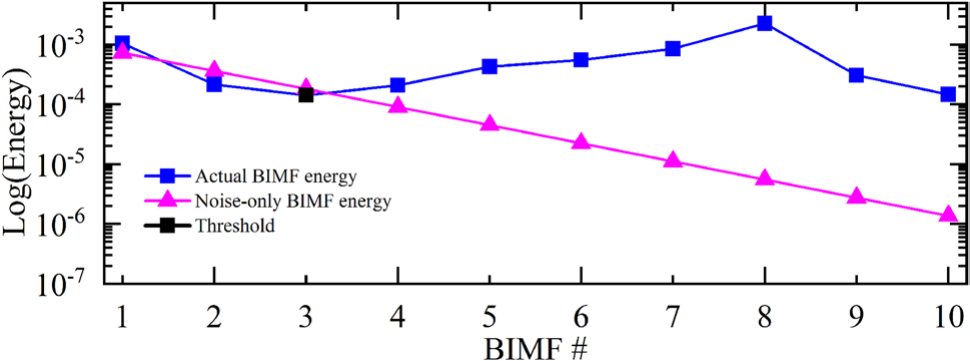
An example of a BIMF energy calculation according to Ref.^56^. The energy of the actual BIMFs is visualized (solid blue line) against the energy of the BIMFs in case they represent noise-only signals (solid magenta line). Information-wise, for the input image $I$ in Fig. 1, the BIMFs 1–3 (*R* = 3) are the least significant. The cut-off is located on the first relevant BIMF (RBIMF) which is # 3 (black squared dot). This can be observed also by evaluating the output of the decomposition. On the other hand, starting from BIMF 4 we observe components having higher energy (which in turn means edges and details that need to be preserved).

To evaluate the relevance of a BIMF (i.e. distinguishing BIMFs carrying information rather than noise) we calculate its energy, *E*, following the approach suggested in Ref.^56^. A sketch of typical BIMF energy curves is depicted in Fig. 2. Similar results have been also observed for the other elaborated images. The energy of the actual BIMFs is visualized (solid blue line) against the energy of the BIMFs in case they would represent noise-only signals (solid magenta line). Those BIMFs whose energy is closer to the energy of noise-only signals are not relevant and can be potentially removed. Information-wise, for the reference image as decomposed in Fig. 2, we have that the first 3 BIMFs are the least significant components. The cut-off is located on BIMF 3 (black squared dot). This can be observed also by evaluating the output of the decomposition. Indeed, from BIMF 4 we observe components having a higher energy (which corresponds to edges and details we need to preserve). Such method enables us to adaptively identify those *R* image components (BIMFs) mostly constituted by noise and remove them accordingly using a nonlinear filtering operator (described in section II.4).

### Nonlinear filtering

The Non-Local Means (NL-means) filter is a nonlinear operator used in image processing to perform image denoising^64^. It has been demonstrated that outperforms a median filter or convolution when trying to simultaneously decrease noise footprint and preserve edges. This technique restores the original image by considering the non-local neighborhoods of a given pixel. The algorithm assigns a higher weight to pixels with similar patches, effectively preserving image structures while reducing noise. The concept of non-local neighborhoods is very useful either in photographic as well as in textured images. It leverages on the redundancy of patterns inside an image and allows a better contribution from different non-adjacent image structures to denoise similar ones. This work utilized the NL-means algorithm as described by Buades and coworkers in Ref.^64^. Mathematically, the NLM algorithm can be described as follows:

Given an input noisy image denoted by *I*, the denoised output image is represented as $I_{NLM}$. Let $N\left( x \right)$ be a neighborhood centered at pixel *x*, which is typically defined as a square or rectangular region of a fixed size *k* around the central pixel, and $W\left( {x,y} \right)$ be the weight that measures the similarity between neighborhoods $N\left( x \right)$ and $N\left( y \right)$. The denoised pixel value $I_{NLM} \left( x \right)$ at location *x* is computed using the following formula:

1 $$ I_{NLM} \left( x \right) \, = \, \left( {{1 \mathord{\left/ {\vphantom {1 {Z\left( x \right)}}} \right. \kern-0pt} {Z\left( x \right)}}} \right) \cdot \sum\nolimits_{y \in \Omega } {I\left( y \right) \cdot W\left( {x, \, y} \right)} \, $$

where Ω is the entire image domain (all pixel locations). $Z\left( x \right)$ is the normalization factor, given by $Z\left( x \right) = \sum\nolimits_{y \in \Omega } {W\left( {x, \, y} \right)} \,$. $I\left( y \right)$ is the pixel value at location *y* in the noisy input image. $W\left( {x,y} \right)$ is the similarity weight between the neighborhoods $N\left( x \right)$ and $N\left( y \right)$.

The formula calculates the denoised pixel value at location *x* as a weighted average of the pixel values in the entire image $\left( {I\left( y \right)} \right)$, where the weight of each pixel is determined by the similarity between the neighborhoods centered at *x* and $y\left( {W\left( {x,y} \right)} \right)$.

The similarity weight $W\left( {x,y} \right)$ in the NLM algorithm is typically determined using a Gaussian kernel function. The Gaussian kernel function is chosen due to its property of assigning higher weights to similar pixel values and lower weights to dissimilar ones. The formula for the similarity weight using the Gaussian kernel function is as follows:

2 $$ W(x,y) = exp\left( {{{ - ||N(x) - N(y)||^{2} } \mathord{\left/ {\vphantom {{ - ||N(x) - N(y)||^{2} } {h^{2} \sigma^{2} }}} \right. \kern-0pt} {h^{2} \sigma^{2} }}} \right) $$

where $W(x,y)$ is the Gaussian similarity weight between neighborhoods $N\left( x \right)$ and $N\left( y \right)$. $||N(x) - N(y)||^{2}$ is the squared Euclidean distance between the two neighborhoods, measuring the similarity in pixel intensity values. *h* is a user-defined parameter that controls the bandwidth of the Gaussian kernel and σ is the standard deviation of the noise in the image.

The Gaussian kernel function provides a measure of similarity between neighborhoods based on the Euclidean distance of their pixel values. When the neighborhoods have similar pixel values, the Gaussian weight is close to 1, indicating high similarity and a higher contribution to the denoised pixel value. Conversely, when the pixel values differ significantly, the Gaussian weight approaches 0, indicating low similarity and a lower contribution to the denoised pixel value.

By adopting the Gaussian kernel function, the NLM algorithm effectively captures the local and non-local self-similarities in the image, allowing it to remove noise while preserving important image structures and textures. The selection of appropriate parameters, such as *h* and *σ*, is crucial to achieving optimal denoising performance and controlling the level of noise reduction and image smoothing. By comparing the neighborhoods of pixels, rather than just their individual grey levels, the algorithm is able to better capture the underlying structures in the image and reduce the impact of noise. This is particularly useful in medical imaging, where noise reduction is essential to accurately identify subtle abnormalities. This fact allows a more robust comparison than classic neighborhood filters^65^. Practically, setting $k = 7$ in the squared neighborhood function, the 7 × 7 similarity window has shown to be large enough to be robust to noise and small enough to take care of details and preserve fine structures. The filtering parameter *h* has been set to $h = 3$. In PACE2.0, the nonlinear filtering is independently applied to the *R*-BIMFs carrying out noise. Once filtered, a reconstruction block then combines the denoised BIMFs with the filtered residual to be further processed using Gamma correction (described in section II.5).

### Gamma correction

Gamma correction techniques make up a family of general Histogram modification techniques obtained by using a varying adaptive parameter γ. The derivation of the transform-based gamma correction (GC) is accomplished through its simple form:

3 $$ T\left( l \right) = l_{\max } \left( {{l \mathord{\left/ {\vphantom {l {l_{\max } }}} \right. \kern-0pt} {l_{\max } }}} \right)^{\gamma } $$

where $l_{max}$ is the maximum intensity of the input. The *l* denotes the intensity of each pixel (*l* $\in$ [0,1]) in the input image is transformed as $T\left( l \right)$ after performing Eq. (3). Many devices used for capturing, printing or displaying the images generally apply a transformation, called power-law^66^, on each pixel of the image that has a nonlinear effect on luminance. As anticipated, the gamma curves illustrated when the gamma value (*γ*) is greater than 1 produce a contrasting effect in comparison to those generated with *γ* values less than 1. It is worth noting that gamma correction tends towards the identity curve when *γ* = 1, which means that there is no alteration to the image. Gamma correction is a non-linear technique employed to modify an image's overall brightness and improve contrast by manipulating the gamma value. In practice, determining the appropriate *γ* value usually involves experimental methods, such as passing a calibration target with a full range of known luminance values through the imaging device. However, in many cases, such calibration is unavailable, or direct access to the imaging device is not feasible. When images are not gamma corrected, they allocate a larger number of bits to represent bright tones, which are indistinguishable to the human eye, and a smaller number of bits for dark tones. This artifact can be removed by means of gamma correction. Hence a solution is required to enhance an image for its gamma values without any knowledge about the imaging device. Here, the gamma correction process is needed to adjust the image quality in different regions in a way that the contrast enhancement is less prone to artifacts that might be generated because of the presence of large over-exposed (very bright) areas. Gamma correction is applied on the reconstructed image (output of method defined in section II.4). The output will be then processed using CLAHE in the subsequent block.

### Contrast limited adaptive histogram equalization—CLAHE

CLAHE represents the generalization of the Adaptive Histogram Equalization^67^. CLAHE segments the input image into a finite number of non-overlapping contextual regions (also called sub-images, tiles, or blocks) and applies histogram equalization to each contextual region. Then it clips the original histogram to a specific value and then redistributes the clipped pixels to each gray level. The technique is already part of the standard PACE algorithm and it has been described in detail in Ref.^13^.

### Multi-objective optimization (MOO)

We iteratively compute the steps (II.2 – II.6) using a combination of different ranges for the control parameters used for HMF (II.2), Energy computation (II.3), Nonlinear Filtering (II.4), Gamma correction (II.5) and CLAHE (II.6). This is organized by using a multi-objective optimization (MOO) technique. Through MOO, we can define an *N*-dimension space of parameters by combining the following different ranges of variability:

- HMF, $g_{H} = [1,1.5,...,2.5]$ and $\beta = [0,1]$ which represents whether or not the filtered BR is used during the reconstruction (8 variations);
- Nonlinear Filtering applied on the *R* least relevant BIMFs computed using Energy as in Ref.^56^ (no variations);
- Gamma Correction^58^ is applied with $\gamma = \left[ {0.5,0.6,...2} \right]$ (16 variations);
- CLAHE (block size [4,4], 2^8^ histogram bins, clip limit $\left[ {0.01 \div 0.02} \right]$). (2 variations);

Overall, the MOO accounts for 256 different permutations of the above-defined parameters. For each iteration a specific combination of parameters is chosen and the corresponding metric scores are calculated being performance objectives. The combination having the joint highest score is selected and the output is generated accordingly.

### Ethical approval

The Ethical Committee at the University Hospital of Messina does not require approval for a work on retrieved and anonymized data. For any information, contact the secretary of the Ethical Committee at the University Hospital of Messina at the contact information posted on its website (https://pre.polime.it/comitato_etico_interaziendale).

## Results and discussions

### Evaluation of the algorithm performance

The performance of PACE2.0 has been benchmarked using patients’ data already available and provided by the Hospitals involved in the testing as well as a CXR database of pulmonary patients of 960 images, which is part of a large public repository (https://www.rsna.org/education/ai-resources-and-training/ai-image-challenge/rsna-pneumonia-detection-challenge-2018)^68–71^. Following the research results presented in literature, we have evaluated the performance of the proposed algorithm considering a set $\Omega$ of well-known reference metrics, CII, ENT, EME^72^ and BRISQUE. For completeness, we have also taken the Total Variation (TV) metric^73^ into account. This metric is commonly used for evaluating noise reduction by measuring smoothness through intensity variations between neighboring pixels. While effective in denoising, PACE2.0 doesn't only reduce noise; it incorporates contrast enhancements like CLAHE and GC, which introduce abrupt intensity changes. These enhancements can increase TV metric values without degrading image quality. Due to its algorithmic complexity and parameter sensitivity, the TV metric becomes unreliable for evaluating PACE2.0, as it performs both denoising and contrast enhancement on the same image, rendering TV an unsuitable measure in this context.

#### Contrast Improvement Index (CII)

According to the literature, the CII is used to measure the increase in contrast generated by an image enhancement method. It is defined as follows^74^:

4 $$ CII = \frac{{C_{processed} }}{{C_{reference} }} $$

where $C_{processed}$ and $C_{reference}$ are the contrast values of the processed and original image, respectively.

The contrast *C* of a region of the gray-level image is represented as^6^:

5 $$ C = \frac{{X_{f} - X_{b} }}{{X_{f} + X_{b} }} $$

$X_{f}$ is the mean luminance value of the foreground and $X_{b}$ is the mean luminance value of the background. As CII rises, the quality of the enhanced image advances.

#### Entropy—ENT

The entropy of an image^6^, is a measure of the randomness characterizing the texture of the image, it quantifies the amount of information content in an image and it is estimated by the histogram of the image considered as a whole:

6 $$ ENT = - \sum {p \cdot \ln \left( p \right)} $$

where *p* is the histogram count for an image segment. ENT serves as an important tool to objectively evaluate and optimize contrast enhancement methods. It allows researchers and practitioners to strike a balance between improving visual appearance and preserving essential information for accurate medical diagnosis and analysis.

#### Effective measure of enhancement—EME

For an image $x\left( {n,m} \right)$ split into $r \times c$ blocks of size $I_{1} \times I_{2}$, the EME is defined as^75^:

7 $$ EME_{r \times c} = \frac{1}{r \times c}\sum\limits_{l = 1}^{r} {\sum\limits_{k = 1}^{c} {\left[ {20\ln \left( {CR_{k,l} } \right)} \right]} } $$

where $\left\{ {k,l} \right\}$ represents the block $B_{k,l}$ considered for the calculations, $EME_{r \times c}$ depends on the image segmentation into $r \times c$ blocks and the contrast $CR_{k,l}$ (as calculated in the block $B_{k,l}$) is defined as^76^:

8 $$ CR_{k,l} = \frac{{{\text{I}}_{max} \{ k,l\} }}{{{\text{I}}_{\min } \{ k,l\} + c}} $$

being ${\text{I}}_{max}$ and ${\text{I}}_{\min }$ are the maximum and minimum intensity levels, respectively, for the image $x\left( {n,m} \right)$ inside $B_{k,l}$. The value *c* is a small constant which is equal to 0.0001 to avoid dividing by 0.

The EME measure is appropriate for images containing attributes such as simple segments (e.g., regular geometric shapes like human body parts), non-periodic patterns within segments, and limited randomness in segments^76^. Moreover, numerous studies on contrast enhancement^77–80^ have utilized EME as an evaluation metric.

#### Blind/referenceless image spatial quality evaluator (BRISQUE)

**Figure 3:**
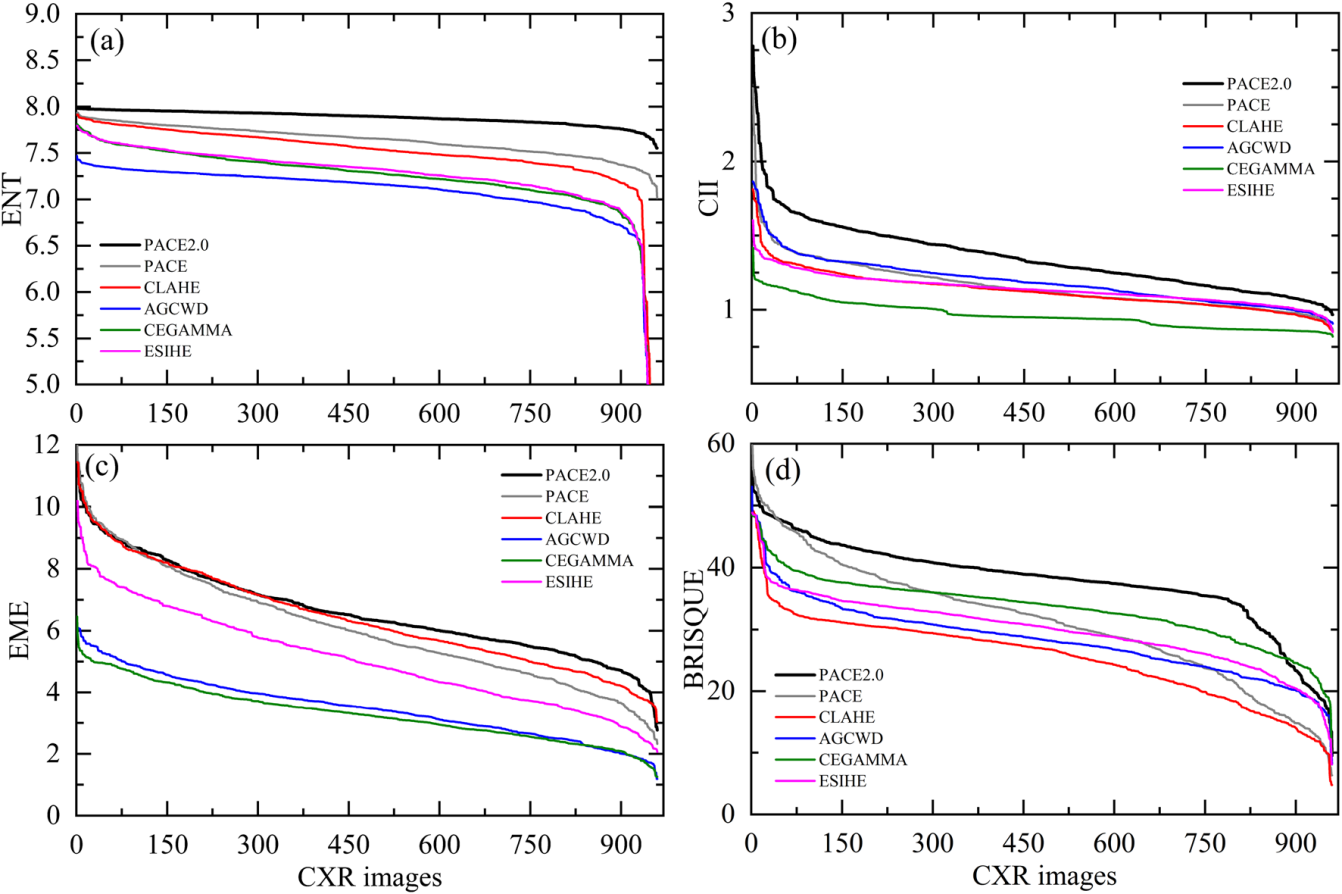
Results as obtained using new PACE2.0 (black line) from the public database (960 CXR images of pneumonia patients processed) and evaluated against PACE (gray line), CLAHE (red line), AGCWD (blue line), CEGAMMA (green line), ESIHE (magenta line) for Entropy (**a**), CII (**b**), EME (**c**), and BRISQUE (**d**), respectively. Our findings demonstrate that PACE2.0 consistently outperforms these methods and provides better performance over the vast majority of the considered cases.

The Blind/Referenceless Image Spatial Quality Evaluator^81^ (BRISQUE) stands out as a pivotal no-reference image quality assessment (IQA) algorithm, adept at appraising the perceptual quality of digital images without the need for a reference image for comparison. Unlike conventional IQA methods^82–84^, which hinge on comparing images against high-quality references, BRISQUE’s significance shines in real-world scenarios where reference images are notably absent, rendering methods like BRISQUE indispensable. In the domain of medical imaging, BRISQUE's potential in image quality assessment has been investigated across diverse medical imaging modalities, including MRI^85,86^, lung CT^87^ scans, and chest X-ray images^88^. This application proves invaluable as it empowers healthcare professionals to ensure that images employed for diagnosis attain requisite quality thresholds, thereby enhancing the reliability of medical assessments. Our evaluation of the BRISQUE algorithm, utilizing our dataset, revealed an upward trajectory indicative of enhanced image quality. This compelling outcome prompted us to incorporate the BRISQUE-generated results into Fig. 3.

#### State-of-the-art methods

The algorithm has showed a quantitative improvement on each of the above considered metrics as compared with state-of-the-art methods for image contrast enhancement listed below:

- Contrast Limited Adaptive Histogram Equalization (CLAHE)^59^
- Adaptive Gamma Correction with Weighting Distribution (AGCWD)^66^
- Contrast Enhanced Gamma Correction (CEGAMMA)^89^
- Exposure-based Sub-Image Histogram Equalization (ESIHE)^90^

which have been used as reference in previous works^91–95^. Figure 3 shows the curves for ENT (a), CII (b), EME (c) and BRISQUE (d), respectively. PACE2.0 (black line) exhibits better performance and outperforms the above methods over the vast majority of the considered cases. In Fig. 3 values are arranged in descending order for better interpretability.

Table 2 presents a summary of the quantitative results obtained for several image enhancement techniques, including CLAHE, AGCWD, CEGAMMA, ESIHE, PACE, and the latest version, PACE2.0. The evaluation was conducted on the same dataset as in Fig. 3, and the best-performing approach was reported for each technique. The results clearly indicate that PACE2.0 is the top-performing method, surpassing the other techniques. Particularly, the introduction of NL-means filtering (described in section II.4) and Gamma Correction (section II.5) have had a remarkable effect on the overall performance when compared to the results obtained by the previous version of the algorithm^13^. When compared to the original CXRs, the results obtained from the three metrics indicate an average increase of 7.5% in ENT, 35% in CII, 95.6% in EME and 13% in BRISQUE, demonstrating an overall improvement in the resulting images.

**Table 2:** Quantitative analysis.

	ENT	CII	EME	BRISQUE
Original CXRs	7.33 +/− 0.47	1.00 +/− 0.00	3.38 +/− 0.95	33.41 +/− 5.48
AGCWD^96^	7.06 +/− 0.44	1.18 +/− 0.15	3.50 +/− 0.99	28.41 +/− 5.89
CEGAMMA^89^	7.22 +/− 0.47	0.96 +/− 0.09	3.30 ± 0.89	33.35 +/− 5.3
ESIHE^90^	7.24 +/− 0.47	1.13 +/− 0.09	5.07 +/− 1.55	29.84 +/− 5.71
CLAHE^59^	7.49 +/− 0.44	1.12 +/− 0.12	6.39 = /-1.59	25.43 +/− 6.9
PACE^13^	7.63 +/− 0.15	1.16 +/− 0.17	6.08 +/− 1.81	31.22 +/− 9.76
**PACE2.0**	**7.88 +/− 0.07**	**1.35 +/− 0.23**	**6.61 +/− 1.44**	**37.87 +/− 6.91**

Performance comparison based on Entropy (ENT), CII, EME and BRISQUE as expressed in average  +/−  standard deviation. Results of 960 CXR images from patients with different pulmonary diseases as evaluated using several state-of-the-art methods. The characters in bold represent the best technique for the chosen metric.

### Artifacts correction

**Figure 4:**
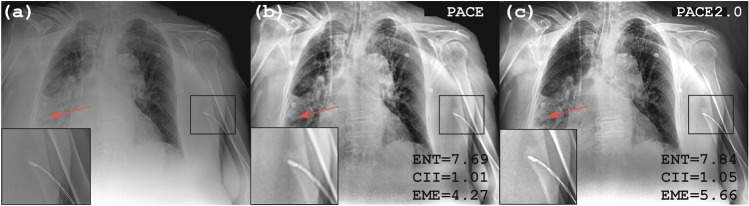
(**a**–**c**) we analyze the impact of non-anatomical object in the enhanced image obtained using PACE (**b**) and PACE2.0 (**c**), respectively. A medical device projecting on the soft tissues on the left arm can be seen (**a**). PACE (**b**) causes blurring of the edges of the object and this can be observed in the magnified area in the bottom left corner. Contrarily, PACE2.0 (**c**) eliminates such an artifact preserving the details of the object. Results obtained using the above defined metrics are visualized (ENT, CII and EME) on the enhanced images Fig. 4 (**b**, **c**).

PACE has been benchmarked by radiologists at two Italian Hospitals, at Research Institutes, and Universities. It has been tested on a wide set of CXRs as part of the Hospital patient database as well as public datasets. These radiographs document a variety of lung diseases, including different types of lung cancer and infection, as well as COVID-19^97^ pneumonia. In the original PACE^13^ algorithm, the post-processing of CXRs allowed a clearer stratification of the subcutaneous soft tissues, a better definition of the cardio-vascular and diaphragmatic profiles, and of the more peripheral bronchovascular patterns. Furthermore, it allowed for clearer recognition and delineation of certain focal changes in the lung tissue (particularly those depicted as bright regions within a low-contrast pulmonary area and which were already apparent in the original images). Conversely, in certain situations: (*i*) it may have underestimated the size of lung abnormalities within regions of low contrast, and (*ii*) it could create a blurring effect when encountering non-anatomical objects (such as prostheses, intravascular devices, etc.), resulting in a loss of detail. In Fig. 4a we observe the original CXR in a patient with a medical device projecting on the soft tissue of the left arm (red arrow). Figure 4b,c illustrate the output using the standard PACE and PACE2.0 implementation, respectively. As it can be seen, the patient in (a) exhibits a relatively large area with a low contrast which prevents the radiologist from delimiting the boundaries of the pleural effusion. (b) PACE does not properly correct this saturation problem and the enhanced image has a suboptimal improvement. Such a result can also be found in other pathological conditions with interstitial-alveolar involvement (‘ground-glass’ pattern). On the other hand, (c) PACE2.0 generates an image with better contrast. In Fig. 4a–c we analyze the impact of non-anatomical object entities in the enhanced image obtained using PACE (b) and PACE2.0 (c), respectively. A medical device projecting on the soft tissue of the left arm can be seen in the native image (a). PACE (b) causes a blurring of the edges of the medical device, shown in the magnified area of the bottom left corner. Contrarily, PACE2.0 generates a contrast-enhanced image and performs a more refined denoising, preserving the details of the object. The improvement is noticeable and also quantitatively represented by better values of the metrics (ENT, CII, and EME) as described above.

### Radiological evaluation

**Figure 5:**
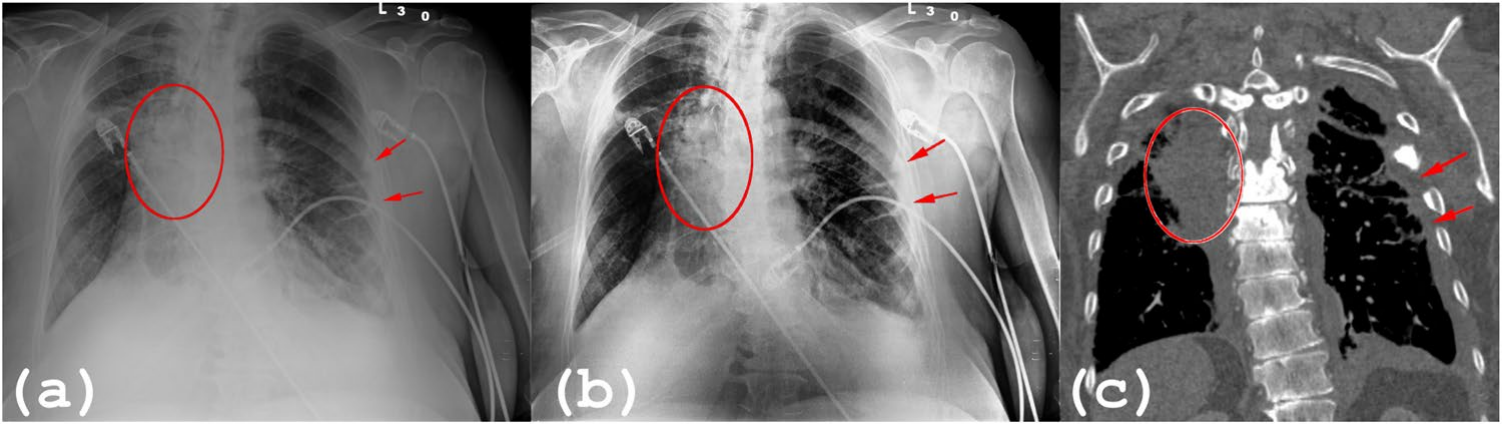
A comparison among (**a**) the original CXR image, (**b**) the enhanced CXR image obtained with PACE2.0, (**c**) the corresponding CT scan. (**a**) on conventional radiography the borders of the lung cancer are not well defined and it is not possible distinguish the lung lesion form adjacent spine (red encircled area). A thickening of the left pleura can be also detected (red arrows). (**b**) On PACE2.0, the image of the right lung mass is easily detectable from spine (red encircled area). In addition, the edges of the left pleural abnormalities are more easily recognizable from lung and ribs (red arrows). The correlation between the PACE2.0 output and the coronal CT reconstruction (**c**) is highly remarkable and indicative of the efficacy of the algorithm as an image enhancement technique.

A qualitative analysis was conducted to assess improvements in diagnostic interpretability in a cohort of 120 patients from both the Italian hospitals involved in the study (Messina and Reggio Calabria). Two expert radiologists evaluated basic chest X-rays and CT scans independently for each patient. In instances of disagreement, the radiologists met to reach a consensus, considering the CT examination results. The use of PACE2.0-enhanced X-rays showed enhanced diagnostic confidence in some cases where lesions were challenging to identify or not visible in the original CXRs. Approximately 10% of the analyzed patients demonstrated such improvements, and their diagnoses were subsequently confirmed by comparing the CT scans. The employment of CT scans was particularly useful in gaining additional insights into these complex cases. To demonstrate the algorithm's improvements in detection accuracy compared to CT scans, we selected certain cases that were considered of particular interest. Figure 5 shows a comparison among the original CXR image (a), the enhanced CXR (b) as computed with PACE, (c) output of PACE2.0, and the corresponding CT scan (d), respectively. In the original CXR image (a), the borders of the lung cancer are not well defined, making it difficult to distinguish the lung lesion from the adjacent spine (red encircled area). Additionally, a thickening of the left pleura can also be detected (red arrows). On the PACE2.0 enhanced image (b), the right lung mass is easily detectable from the spine (red encircled area). Moreover, the edges of the left pleural abnormalities are more easily recognizable from the lung and ribs (red arrows). In addition, the edges of the left pleural abnormalities are more easily recognizable from lung and ribs (red arrows). (c) The correlation between the PACE2.0 output and the coronal CT reconstruction is highly remarkable and indicative of the efficacy of PACE2.0 as an image enhancement technique.

**Figure 6:**
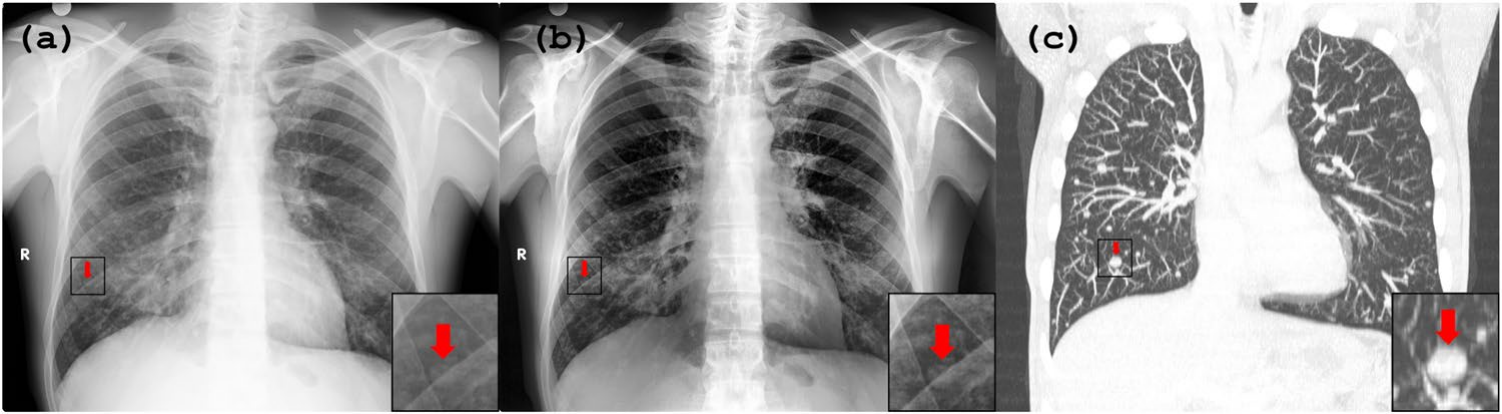
The patient in question has multiple lung metastases resulting from malignant paraganglioma. The image set comprises (**a**) the original anteroposterior CXR, (b) the image obtained after applying PACE2.0, and (**c**) the corresponding CT image. The initial chest radiograph displays haziness in both lungs, with multiple small nodules that are poorly defined (red arrow), resulting in low diagnostic confidence. In contrast, the nodules are more apparent in (**b**), the PACE2.0-processed image, where there is a noticeable improvement in the edge definition of one nodule (red arrow), which can be seen more clearly in the insets. The apparent difference of position of nodule between the X-ray image (**a** and **b**) and CT scan (**c**) is due to the different position of the patient during acquisition of images (supine on CT and in standing position on x-ray) and consequent differences in inspiration depth. The reconstructed coronal chest CT image (**c**) confirms the presence of multiple nodular (red nodule) metastases in both lungs.

Figure 6 presents a case study of a patient with multiple lung metastases from malignant paraganglioma. The set includes (a) the original anteroposterior CXR, (b) the result obtained using PACE2.0, and (c) the corresponding CT image. The initial chest radiograph displays haziness in both lungs, and multiple small nodules are poorly defined (red arrow), resulting in low diagnostic confidence. However, the insets in the PACE2.0-enhanced image demonstrate the visibly improved edge definition of one nodule (red arrow), indicating that the approach can enhance specific regions of an image. The apparent difference of position of nodule between the X-ray image ((a) and (b)) and CT scan (c) is due to the different position of the patient during acquisition of images (supine on CT and in standing position on X-ray) and consequent differences in inspiration depth. Different lung air distension causes the apparent repositioning of nodule and reflects a result of the technical and physiological disparities associated with the imaging process in the two different scenarios. The reconstructed coronal chest CT image (c) confirms (red arrow) the presence of multiple nodular metastases in both lungs.

**Figure 7:**
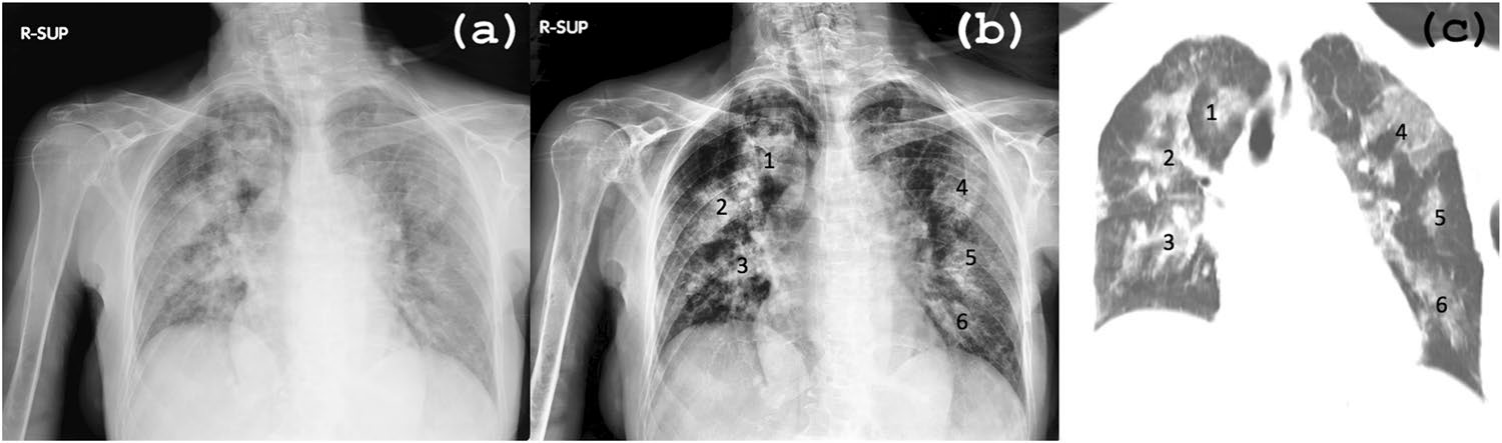
Female patient diagnosed with bilateral pneumonia. The image set includes (**a**) the original CXR image, (**b**) the output using PACE2.0, and (**c**) the corresponding CT scan. The PACE2.0-processed output image allows for better definition of the extension of the disease in comparison with the native CXR due to the enhanced contrast between the edges of the pneumonia and the normal lung tissue. The correlation analysis shows a perfect correspondence between the PACE2.0 output and the coronal CT reconstruction (**c**), with both examinations detecting six areas of pneumonia. This finding highlights the accuracy of PACE2.0 in detecting and characterizing pulmonary abnormalities, particularly in low-contrast images.

In Fig. 7, we present an example of a female patient diagnosed with bilateral pneumonia. The image set consists of (a) the original image, (b) the PACE2.0-processed output, and (c) the corresponding CT scan. Despite the low contrast of the original image, which makes it difficult to identify multiple lesions (particularly those marked as 4, 5, and 6 in the left lung), the PACE2.0 output image significantly improves the definition of the disease. This is due to the enhanced contrast between the edges of the pneumonia and the normal lung tissue. As a result, the algorithm achieves superior identification of all the lesions in both lungs, their location, and their extension. The correlation between the PACE2.0 output and the coronal CT reconstruction (c) is noteworthy. In both examinations, six areas of pneumonia are visible, demonstrating the perfect correspondence between the two imaging modalities. This finding underscores the accuracy of the PACE2.0 algorithm in detecting and characterizing pulmonary abnormalities.

**Figure 8:**
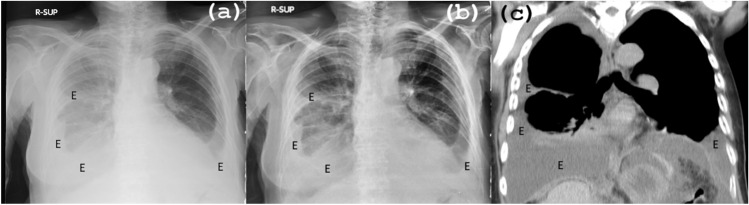
Male patient with bilateral pleural effusion, comparison among (**a**) original CXR image, (**b**) output using PACE2.0 and CT scan (**c**). The effusions are better visible on the resulting image with PACE2.0 due to more defined borders between lungs and pleural opacities. Correlation with a coronal CT reconstruction demonstrates the perfect correspondence between the two examinations. E = pleural effusion.

In Fig. 8, a male patient with bilateral pleural effusion is presented, and the corresponding original CXR (a), PACE2.0 image (b), and CT scan (c) are displayed. In the original image (a), the pleural effusions are labeled as E, but the borders between the lungs and the pleural opacities are not well-defined. In contrast, the PACE2.0 image (b) clearly displays the effusions due to the enhanced contrast and improved borders. Correlation with the coronal CT reconstruction (c) validates the accuracy of the findings on both examinations.

**Figure 9:**
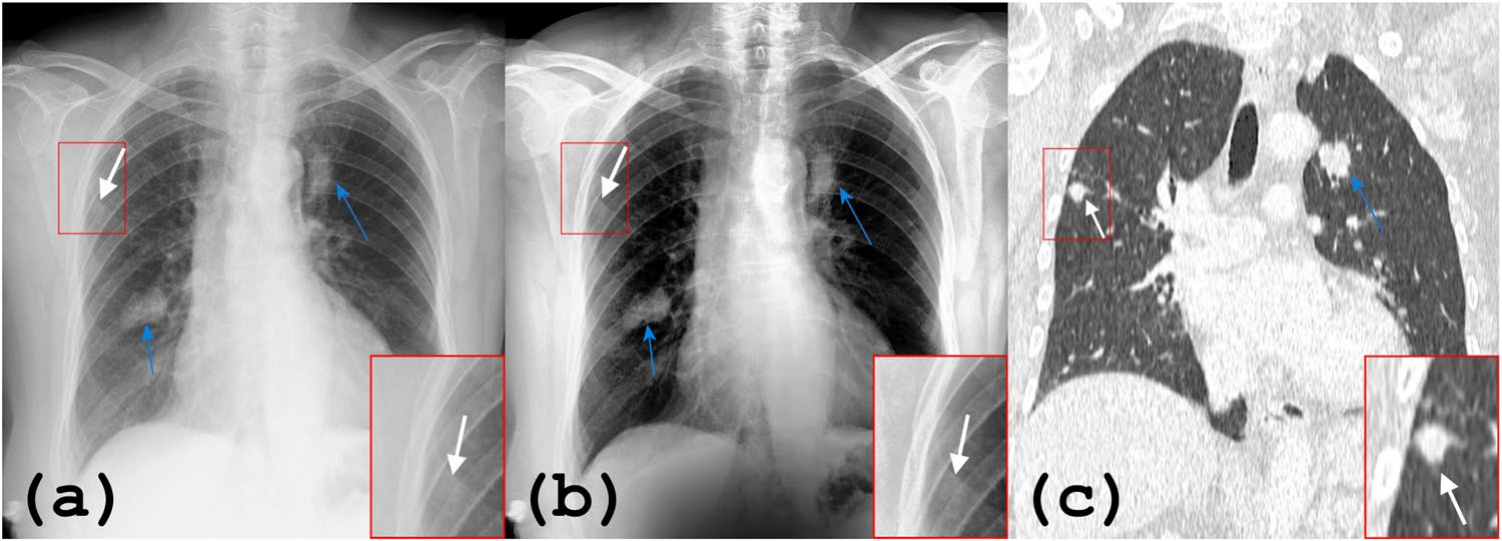
Chest radiography (**a**), resulting output using PACE2.0 (**b**) and (**c**) coronal multiplanar reconstruction of high-resolution computed tomography (CT) of a female patient with high-grade breast cancer. (**a**) The CXR reveals two metastases: one in the left upper lobe (indicated by a blue arrow) and one in the middle lobe of the right lung (also indicated by a blue arrow). However, the PACE2.0-enhanced image (**b**) reveals an additional small metastasis in the right upper lobe, which was previously obscured in (**a**) by a rib (indicated by a white arrow). The coronal CT (**c**) scan reconstruction (different plane) conclusively confirms the presence of the small lesion.

Figure 9 illustrates a case of a female patient with multiple nodular lung metastases from breast cancer. The original CXR (a), the one elaborated with PACE2.0 (b), and (c) the coronal multiplanar reconstruction of computed tomography (CT) are presented. The CXR shows two metastases one in the left upper lobe (blue arrow) and one in the middle lobe of the right lung (blue arrow), respectively. On the PACE2.0-enhanced image another small metastasis in the right upper lobe overlapped by a rib can be detected (white arrow). A retrospective evaluation of the original CXR (a) shows that the above mentioned lesion is poorly identifiable when compared against PACE2.0-processed CXR (b). Ultimately, coronal CT (c) scan reconstruction (different plane) confirms the presence of the small lesion as can be shown in the insets on the bottom right corners for (a), (b) and (c).

### Learning classification enhancement by using PACE 2.0

DL with convolutional neural network CNNs has a lot of success stories in the area of image recognition and classification^98^ with an increasing number of CAD (computer-aided design) systems to diagnose^99,100^ However, their application to pulmonary diseases remains bounded to specific cases^101,102^. Currently, most of the key problems are the limited size of the public dataset and the acquisition constraints^103^, which in turn negatively impact image quality as well as the performance of DL algorithms for classification^104^. Indeed, a large number of high-quality training samples are more capable of producing successful models. According to Koo and Cha^105^, image pre-processing is important when training CNN models, because it can effectively boost their performance in classification. Hence, research into the relationship between image enhancement and DL models is pivotal^106–112^. Concerning image quality, Munadi et al.^113^ proved through experiments that using unsharp masking (UM) image enhancement algorithms or high-frequency emphasis filtering (HEF) on chest X-ray images of tuberculosis patients can effectively improve the judgment ability of CNNs. In their analysis, EfficientNet^113^ with Unsharp masking image enhancement achieved 89.92% accuracy on the Shenzeng dataset for pulmonary diseases^114^. Msnoda et al.^115^ implemented ResNet^116^, GoogLeNet^117^, and AlexNet^118^ with an extra Spatial Pyramid Pooling (SPP)^115^ layer. Out of the architectures that were implemented, GoogLeNet was found to have the highest classification accuracy of 97%. Furthermore, the performance of this architecture was further enhanced by incorporating the SPP (Spatial Pyramid Pooling) layer, which improved the accuracy to 98%. Overall, the HEF algorithm has proven to perform well on medical images^12^. Because of that, we evaluated the effect of the PACE2.0 algorithm as a pre-processing step for the performance of a state-of-the-art DL architecture, DenseNet-121, which has not been fully investigated so far, even in the most recent literature. We will assess the effect of different inputs (comparing results against original, enhanced CXRs using several state-of-the-art methods and PACE2.0) on the use of a pre-trained DL architecture to detect patients having pneumonia from normal (i.e. healthy) subjects. A balanced training dataset has been built from RSNA^68^ consisting of 21,832 CXR images of pneumonia and normal patients, using a 10% ratio for validation. Then, a balanced test set of chest radiographies (a collection of 960 images each for pneumonia and for healthy patients) has been used for evaluating performance by comparing original CXR images (no enhancement), against enhanced CXRs using CLAHE, ESIHE CEGAMMA, AGCWD and PACE2.0, respectively.

**Figure 10:**
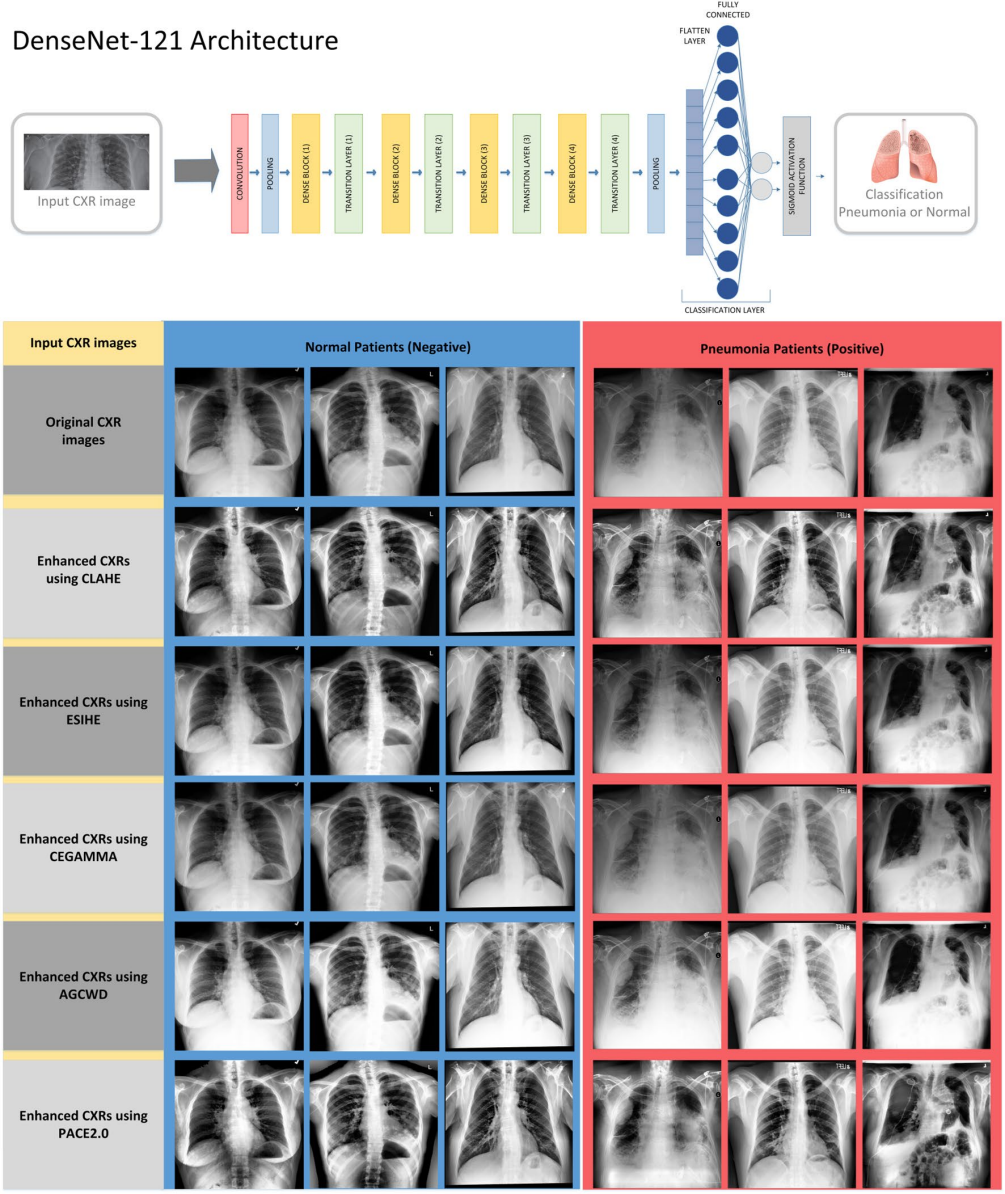
(Top) The deep learning-based architecture for pneumonia detection using DenseNet-121. (Bottom) Some examples of testing images during experiment considering different types of inputs: original CXR images (no enhancement), enhanced CXRs using CLAHE, ESIHE CEGAMMA, AGCWD and PACE2.0, respectively.

In Fig. 10 (top), we illustrate the DL architecture for detecting pneumonia using a pre-trained DenseNet-121 model^50^. The model was pre-trained on ImageNet^118^. The input to the model is a grayscale CXR image that has been rescaled to a dimension of 224 × 224 × 1 byte (height, width, and bit depth). For transfer learning, we remove the original classification layer of DenseNet-121 model (last layer) and replace it with a new fully connected layer. The new Fully Connected layer of the network is then re-trained to classify the extracted features from the convolutional layers block to the target classes, which are either chest images of healthy (normal) or pneumonia patients. Figure 10 (bottom) displays examples of the different images that were considered as input to the DL model. These input images include the original CXRs with no enhancement, as well as the enhanced CXRs using CLAHE, ESIHE, CEGAMMA, AGCWD, and PACE2.0. We analyze the classification performance considering the different inputs and against several metrics whose results are presented in Table 3. This table summarizes how our algorithm increases the classification capability of a DenseNet-121 model using transfer learning. Such evaluation measures are widely known^119^ and are represented by accuracy, recall, precision, F_1_ score and the area under curve (AUC) of a receiver operator characteristic (ROC), which is still studied for assessing imaging tests^120^. The PACE2.0 once applied to feed the DenseNet-121 model could notably achieve a high prediction accuracy (94%), recall (97%). These findings demonstrate the effectiveness of PACE2.0 in enhancing CXR images to improve the detection accuracy of the DL model for pneumonia.

**Table 3:** Accuracy, recall, precision, F_1_ and AUC scores as obtained using transfer learning for image classification (we used a pre-trained DenseNet-121 model).

Method	Accuracy	Recall	Precision	F_1_ score	AUC
Original CXR	0.808	0.893	0.763	0.823	0.859
CLAHE	0.814	0.897	0.769	0.828	0.864
ESIHE	0.904	0.949	0.871	0.908	0.915
CEGAMMA	0.909	0.952	0.877	0.913	0.921
AGCWD	0.924	0.961	0.895	0.927	0.939
**PACE2.0**	**0.947**	**0.974**	**0.925**	**0.949**	**0.960**

Comparison of the metrics against baseline original CXRs, CLAHE, ESIHE, CEGAMMA, AGCWD and PACE2.0 algorithms, respectively. Significant values are in [bold].

In Fig. A1 (supplementary material) we also present the plot of the ROC of the DenseNet-121 model considering different input configurations. The ROC curve is a powerful tool to analyze the tradeoff between the true positive rate and the false positive rate across a full range of possible thresholds.

Comparing the AUC (Area Under Curve) values of the ROC, unprocessed CXRs (yellow line) and CLAHE-processed images (light green line) exhibit the lowest values at 0.859 and 0.864, respectively. Conversely, AGCWD (purple line) and PACE2.0 (violet line) achieve the highest prediction accuracy, with AUC values of 0.939 and 0.960, respectively. These results emphasize the impact of image processing techniques on the classifier's performance and highlight the effectiveness of AGCWD and PACE2.0 in enhancing the model's accuracy.

## Summary and conclusions

This work expands the opportunity to consider reliable state-of-the-art enhancement techniques to be performed before medical image interpretation with the development of PACE2.0. The performance is evaluated on CXR images to support and improve the capability to pneumonia detection either by humans or DL algorithms (DensetNet-121 in this work). A quantitative improvement has been quantified with three well-known metrics, achieving an increase of 7.5% in ENT, 35% in CII, 95.6% in EME and 13% in BRISQUE, respectively, when compared against original CXRs and is generally higher than other image-enhancement techniques such as CLAHE, AGCWD, CEGAMMA, and ESIHE. Overall, PACE2.0 preserves the input image details more accurately, yielding processed images with better contrast enhancement, reduced brightness inhomogeneities, and mitigating the occurrence of artifacts. Ultimately, this tool can support the clinical assistance of patients by enhancing the readability of CXRs for the monitoring of different patients affected by pulmonary lesions, including COVID-19 patients as well as patients in intensive care units. PACE2.0 can be also used as a support for clinical activities either in poor regions where CT is not available or hospitals and in under-developed countries. In addition, it can be potentially used as a tool for data preprocessing or data augmentation in machine/deep learning approaches, such as segmentation and classification, with a possible application in the early diagnosis of pulmonary diseases^39,121,122^. Future works will explore the incorporation of statistical data to further validate and substantiate its artefact mitigation capabilities. Additionally, the investigation will encompass the integration of supplementary image enhancement techniques, along with rib suppression methods, to achieve a more pronounced enhancement effect on chest radiographs.

### Supplementary Information

Supplementary Information.

## Data Availability

The chest radiographies (Dicom images) collected at the two Italian hospitals, have been acquired using a Fujifilm FCR x-ray image scanner, manufacturer FUJIFILM Corporation and are available from the corresponding author on reasonable request. The other data used are part of the Radiological Society for North America (RSNA) challenge and are available on the Kaggle repository (https://www.rsna.org/education/ai-resources-and-training/ai-image-challenge/RSNA-Pneumonia-Detection-Challenge-2018). The datasets used and/or analysed during the current study available from the corresponding author on reasonable request.

## References

[1] Troeger C (2018). Estimates of the global, regional, and national morbidity, mortality, and aetiologies of lower respiratory infections in 195 countries, 1990–2016: A systematic analysis for the Global Burden of Disease Study 2016. Lancet Infect. Dis..

[2] Kroft LJM (2019). Added value of ultra–low-dose computed tomography, dose equivalent to chest X-ray radiography, for diagnosing chest pathology. J. Thorac. Imaging.

[3] Masjedi H (2020). European trends in radiology: Investigating factors affecting the number of examinations and the effective dose. Radiol. Med..

[4] Delrue, L. *et al.* Difficulties in the interpretation of chest radiography. In (eds. Coche, E. E., Ghaye, B., de Mey, J. & Duyck, P.) 27–49 (Springer Berlin Heidelberg, 2011). 10.1007/978-3-540-79942-9_2.

[5] Rahman T (2021). Exploring the effect of image enhancement techniques on COVID-19 detection using chest X-ray images. Comput. Biol. Med..

[6] Wu, S., Zhu, Q., Yang, Y. & Xie, Y. Feature and contrast enhancement of mammographic image based on multiscale analysis and morphology. In *2013 IEEE International Conference on Information and Automation (ICIA), 26–28 Aug 2013,* 521–526 (IEEE, 2013). 10.1109/ICInfA.2013.6720354. 10.1155/2013/716948 PMC3876670 24416072

[7] Sun X, Xu Q, Zhu L (2019). An effective Gaussian fitting approach for image contrast enhancement. IEEE Access.

[8] Ortiz-Jaramillo B, Kumcu A, Platisa L, Philips W (2018). Content-aware contrast ratio measure for images. Signal Process. Image Commun..

[9] Prasad GR (2016). An algorithmic approach towards chest X-ray image enhancement by global histogram equalization. IRA-Int. J. Technol. Eng..

[10] Kaur P, Singh G, Kaur P (2018). A review of denoising medical images using machine learning approaches. Curr. Med. Imaging Rev..

[11] Michael P, Yoon H-J, Hahn HK, Mazurowski MA (2020). Survey of image denoising methods for medical image classification. Medical Imaging 2020: Computer-Aided Diagnosis.

[12] Rajab MI, Eskandar AA (2011). Enhancement of radiographic images in patients with lung nodules. Thorac. Cancer.

[13] Siracusano G (2020). Pipeline for advanced contrast enhancement (PACE) of chest X-ray in evaluating COVID-19 patients by combining bidimensional empirical mode decomposition and contrast limited adaptive histogram equalization (CLAHE). Sustainability.

[14] Chen Y (2019). Bone suppression of chest radiographs with cascaded convolutional networks in wavelet domain. IEEE Access.

[15] Aidoo AY, Wilson M, Botchway GA (2019). Chest radiograph image enhancement with wavelet decomposition and morphological operations. TELKOMNIKA Telecommun. Comput. Electron. Control..

[16] Na’am J, Santony J, Yuhandri Y, Sumijan S, Nurcahyo GW (2018). Enlarge medical image using line-column interpolation (LCI) method. Int. J. Electr. Comput. Eng..

[17] Kumar R, Bhattacharya S, Murmu G (2021). Exploring optimality of piecewise polynomial interpolation functions for lung field modeling in 2D chest X-ray images. Front. Phys..

[18] Vieira P, Sousa O, Magalhães D, Rabêlo R, Silva R (2021). Detecting pulmonary diseases using deep features in X-ray images. Pattern Recognit..

[19] Fonseca, A. U. *et al.* X-ray image enhancement: A technique combination approach. In *2019 IEEE 31st International Conference on Tools with Artificial Intelligence (ICTAI)* 1686–1690 (IEEE, 2019). 10.1109/ICTAI.2019.00248.

[20] Mahmood A, Khan SA, Hussain S, Almaghayreh EM (2019). An adaptive image contrast enhancement technique for low-contrast images. IEEE Access.

[21] Bakthula R, Agarwal S (2018). Radiographic X-ray images enhancement with edge preservation using singular value decomposition. Int. J. Comput. Phys. Ser..

[22] Xue, Z. *et al.* Foreign object detection in chest X-rays. In *2015 IEEE International Conference on Bioinformatics and Biomedicine (BIBM)* 956–961 (IEEE, 2015). 10.1109/BIBM.2015.7359812.

[23] Koonsanit, K., Thongvigitmanee, S., Pongnapang, N. & Thajchayapong, P. Image enhancement on digital x-ray images using N-CLAHE. In *2017 10th Biomedical Engineering International Conference (BMEiCON)* 1–4 (IEEE, 2017). 10.1109/BMEiCON.2017.8229130.

[24] Jena, D. & Pradhan, N. Image processing techniques for chest radiography enhancements and pneumonia detection. In *2021 International Conference on Emerging Techniques in Computational Intelligence (ICETCI)* 1–5 (IEEE, 2021). 10.1109/ICETCI51973.2021.9574077.

[25] Amar Tej G, Shah PK (2015). Efficient quality analysis and enhancement of MRI image using Filters and Wavelets. Int. J. Adv. Res. Comput. Commun. Eng..

[26] Zhang M, Mou X (2010). Nonlinear multi-scale contrast enhancement for X-ray chest radiography. Hsi-An Chiao Tung Ta Hsueh/J. Xi’an Jiaotong Univ..

[27] Anand, S. Medical image enhancement using edge information-based methods. In *Computational Tools and Techniques for Biomedical Signal Processing* 123–148 (2017). 10.4018/978-1-5225-0660-7.ch006.

[28] Chang Y, Jung C, Ke P, Song H, Hwang J (2018). Automatic contrast-limited adaptive histogram equalization with dual gamma correction. IEEE Access.

[29] Joseph J, Sivaraman J, Periyasamy R, Simi VR (2017). An objective method to identify optimum clip-limit and histogram specification of contrast limited adaptive histogram equalization for MR images. Biocybern. Biomed. Eng..

[30] Jenifer S, Parasuraman S, Kadirvelu A (2016). Contrast enhancement and brightness preserving of digital mammograms using fuzzy clipped contrast-limited adaptive histogram equalization algorithm. Appl. Soft Comput..

[31] Salem N, Malik H, Shams A (2019). Medical image enhancement based on histogram algorithms. Procedia Comput. Sci..

[32] Alavijeh F, Mahdavi-Nasab H (2015). Multi-scale morphological image enhancement of chest radiographs by a hybrid scheme. J. Med. Signals Sens..

[33] Chen S, Zhong S, Yao L, Shang Y, Suzuki K (2016). Enhancement of chest radiographs obtained in the intensive care unit through bone suppression and consistent processing. Phys. Med. Biol..

[34] Khan MF, Khan E, Abbasi ZA (2014). Segment dependent dynamic multi-histogram equalization for image contrast enhancement. Digit. Signal Process..

[35] Bhandari AK, Subramani B, Veluchamy M (2022). Multi-exposure optimized contrast and brightness balance color image enhancement. Digit. Signal Process..

[36] Rao BS (2020). Dynamic histogram equalization for contrast enhancement for digital images. Appl. Soft Comput..

[37] Khan MF (2020). Fuzzy-based histogram partitioning for Bi-histogram equalisation of low contrast images. IEEE Access.

[38] Nefoussi, S., Amamra, A. & Amarouche, I. A. A Comparative study of chest X-ray image enhancement techniques for pneumonia recognition. In *Lecture Notes in Networks and Systems* 276–288 (2021). 10.1007/978-3-030-69418-0_25.

[39] Lv D (2021). A cascade-SEME network for COVID-19 detection in chest x-ray images. Med. Phys..

[40] Mukherjee, J., Sikdar, B., Chakrabarti, A., Kar, M. & Das, S. A novel technique for contrast enhancement of chest X-ray images based on bio-inspired meta-heuristics. In *Advances in Intelligent Systems and Computing* 71–93 (2018). 10.1007/978-981-10-8180-4_5.

[41] Tschuchnig ME, Gadermayr M, Haber P, Lampoltshammer TJ, Leopold H, Mayr M (2022). Anomaly detection in medical imaging: A mini review. Data Science—Analytics and Applications.

[42] Minaee S, Kafieh R, Sonka M, Yazdani S, Jamalipour Soufi G (2020). Deep-COVID: Predicting COVID-19 from chest X-ray images using deep transfer learning. Med. Image Anal..

[43] Chowdhury NK, Kabir MA, Rahman MM, Rezoana N (2021). ECOVNet: A highly effective ensemble based deep learning model for detecting COVID-19. PeerJ Comput. Sci..

[44] Qureshi MA, Beghdadi A, Deriche M (2017). Towards the design of a consistent image contrast enhancement evaluation measure. Signal Process. Image Commun..

[45] Wang X, Chen L (2017). An effective histogram modification scheme for image contrast enhancement. Signal Process. Image Commun..

[46] Campbell J, Pyer M, Rogers S, Walter D, Reddy R (2014). Enabling patients with respiratory symptoms to access chest X-rays on demand: The experience of the walk-in service in Corby, UK. J. Public Health (Bangkok).

[47] Cao AMY, Choy JP, Mohanakrishnan LN, Bain RF, van Driel ML (2013). Chest radiographs for acute lower respiratory tract infections. Cochrane Database Syst. Rev..

[48] Walz-Flannigan AI, Brossoit KJ, Magnuson DJ, Schueler BA (2018). Pictorial review of digital radiography artifacts. RadioGraphics.

[49] Walz-Flannigan A, Magnuson D, Erickson D, Schueler B (2012). Artifacts in digital radiography. Am. J. Roentgenol..

[50] Huang, G., Liu, Z., Van Der Maaten, L. & Weinberger, K. Q. Densely connected convolutional networks. In *2017 IEEE Conference on Computer Vision and Pattern Recognition (CVPR)* 2261–2269 (IEEE, 2017). 10.1109/CVPR.2017.243.

[51] Shih G (2019). Augmenting the National Institutes of Health Chest Radiograph Dataset with expert annotations of possible pneumonia. Radiol. Artif. Intell..

[52] Bhuiyan, S. M. A., Adhami, R. R. & Khan, J. F. A novel approach of fast and adaptive bidimensional empirical mode decomposition. In *2008 IEEE International Conference on Acoustics, Speech and Signal Processing* 1313–1316 (IEEE, 2008). 10.1109/ICASSP.2008.4517859.

[53] Wielgus, M., Antoniewicz, A., Bartyś, M. & Putz, B. Fast and adaptive bidimensional empirical mode decomposition for the real-time video fusion. In *15th International Conference on Information Fusion, FUSION 2012* 649–654 (2012).

[54] Pitas I, Venetsanopoulos AN (1990). Nonlinear Digital Filters Nonlinear Digital Filters.

[55] Firdaus Zakaria, M., Ibrahim, H. & Azmin Suandi, S. A review: Image compensation techniques. In *2010 2nd International Conference on Computer Engineering and Technology* V7-404-V7-408 (IEEE, 2010). 10.1109/ICCET.2010.5485499.

[56] Flandrin, P., Gonçalvès, P. & Rilling, G. EMD equivalent filter banks, from interpretations to applications. 99–116 (2014). 10.1142/9789814508247_0005.

[57] Zhu Y, Huang C (2012). An improved median filtering algorithm for image noise reduction. Phys. Procedia.

[58] Bull DR, Zhang F, Bull DR, Zhang F (2021). Chapter 4 - Digital picture formats and representations. Intelligent Image and Video Compression.

[59] Zuiderveld K (1994). Contrast limited adaptive histogram equalization. Graphics Gems.

[60] Dutta P, Saha S (2017). Fusion of expression values and protein interaction information using multi-objective optimization for improving gene clustering. Comput. Biol. Med..

[61] Yang X-S, Yang X-S (2014). Multi-objective optimization. Nature-Inspired Optimization Algorithms.

[62] Bhuiyan SMA, Adhami RR, Khan JF (2008). Fast and adaptive bidimensional empirical mode decomposition using order-statistics filter based envelope estimation. EURASIP J. Adv. Signal Process..

[63] Palkar, P. M., Udupi, V. R. & Patil, S. A. A review on bidimensional empirical mode decomposition: A novel strategy for image decomposition. In *2017 International Conference on Energy, Communication, Data Analytics and Soft Computing (ICECDS)* 1098–1100 (IEEE, 2017). 10.1109/ICECDS.2017.8389610.

[64] Buades, A., Coll, B. & Morel, J. M. A non-local algorithm for image denoising. In *Proceedings - 2005 IEEE Computer Society Conference on Computer Vision and Pattern Recognition, CVPR 2005* **II**, (2005).

[65] Lee J-S (1983). Digital image smoothing and the sigma filter. Comput. Vis. Graph. Image Process..

[66] Rahman S, Rahman MM, Abdullah-Al-Wadud M, Al-Quaderi GD, Shoyaib M (2016). An adaptive gamma correction for image enhancement. EURASIP J. Image Video Process..

[67] Zimmerman JB (1988). An evaluation of the effectiveness of adaptive histogram equalization for contrast enhancement. IEEE Trans. Med. Imaging.

[68] Wang, X. *et al.* ChestX-ray: Hospital-scale chest X-ray database and benchmarks on weakly supervised classification and localization of common thorax diseases. In *Advances in Computer Vision and Pattern Recognition* 369–392 (2019). 10.1007/978-3-030-13969-8_18.

[69] Kelly B (2012). The chest radiograph. Ulster Med. J..

[70] Franquet T (2018). Imaging of community-acquired pneumonia. J. Thorac. Imaging.

[71] Rui, P. & Kang, K. National Hospital Ambulatory Medical Care Survey. *Natl. Cent. Heal. Stat.* (2015).

[72] Panetta K, Samani A, Agaian S (2014). Choosing the optimal spatial domain measure of enhancement for mammogram images. Int. J. Biomed. Imaging.

[73] Buades A, Coll B, Morel JM (2005). A review of image denoising algorithms, with a new one. Multiscale Model. Simul..

[74] Ema T, Doi K, Nishikawa RM, Jiang Y, Papaioannou J (1995). Image feature analysis and computer-aided diagnosis in mammography: Reduction of false-positive clustered microcalcifications using local edge-gradient analysis. Med. Phys..

[75] Agaian SS, Silver B, Panetta KA (2007). Transform coefficient histogram-based image enhancement algorithms using contrast entropy. IEEE Trans. Image Process..

[76] Gupta S, Porwal R (2016). Appropriate contrast enhancement measures for brain and breast cancer images. Int. J. Biomed. Imaging.

[77] Sundaram M, Ramar K, Arumugam N, Prabin G (2011). Histogram modified local contrast enhancement for mammogram images. Appl. Soft Comput..

[78] Arici T, Dikbas S, Altunbasak Y (2009). A histogram modification framework and its application for image contrast enhancement. IEEE Trans. Image Process..

[79] Lee C, Lee C, Kim C-S (2013). Contrast enhancement based on layered difference representation of 2D histograms. IEEE Trans. Image Process..

[80] Chen S-D (2012). A new image quality measure for assessment of histogram equalization-based contrast enhancement techniques. Digit. Signal Process..

[81] Mittal A, Moorthy AK, Bovik AC (2012). No-reference image quality assessment in the spatial domain. IEEE Trans. Image Process..

[82] Shruti S, Deshpande AM (2014). Image quality assessment techniques: An overview. Int. J. Eng. Res. Technol..

[83] Barman SA, Welikala RA, Rudnicka AR, Owen CG (2019). Image quality assessment. Computational Retinal Image Analysis.

[84] Liu A, Lin W, Narwaria M (2012). Image quality assessment based on gradient similarity. IEEE Trans. Image Process..

[85] Zhang Z (2018). Can signal-to-noise ratio perform as a baseline indicator for medical image quality assessment. IEEE Access.

[86] Chow LS, Rajagopal H (2017). Modified-BRISQUE as no reference image quality assessment for structural MR images. Magn. Reson. Imaging.

[87] Ibrahim RW, Jalab HA, Karim FK, Alabdulkreem E, Ayub MN (2022). A medical image enhancement based on generalized class of fractional partial differential equations. Quant. Imaging Med. Surg..

[88] Aldoury RS, Al-Saidi NMG, Ibrahim RW, Kahtan H (2023). A new X-ray images enhancement method using a class of fractional differential equation. MethodsX.

[89] Jiang G (2015). Image contrast enhancement with brightness preservation using an optimal gamma correction and weighted sum approach. J. Mod. Opt..

[90] Singh K, Kapoor R, Sinha SK (2015). Enhancement of low exposure images via recursive histogram equalization algorithms. Optik (Stuttg)..

[91] Singh N, Kaur L, Singh K (2019). Histogram equalization techniques for enhancement of low radiance retinal images for early detection of diabetic retinopathy. Eng. Sci. Technol. Int. J..

[92] Al-Ameen Z (2018). Expeditious contrast enhancement for grayscale images using a new swift algorithm. Stat. Optim. Inf. Comput..

[93] Singh K, Vishwakarma DK, Walia GS, Kapoor R (2016). Contrast enhancement via texture region based histogram equalization. J. Mod. Opt..

[94] Wong CY (2016). Image contrast enhancement using histogram equalization with maximum intensity coverage. J. Mod. Opt..

[95] Singh K, Kapoor R (2014). Image enhancement via median-mean based sub-image-clipped histogram equalization. Optik (Stuttg)..

[96] Huang S-C, Cheng F-C, Chiu Y-S (2013). Efficient contrast enhancement using adaptive gamma correction with weighting distribution. IEEE Trans. Image Process..

[97] Kermany DS (2018). Identifying medical diagnoses and treatable diseases by image-based deep learning. Cell.

[98] Stephen O, Sain M, Maduh UJ, Jeong D-U (2019). An efficient deep learning approach to pneumonia classification in healthcare. J. Healthc. Eng..

[99] Apostolopoulos ID, Mpesiana TA (2020). Covid-19: Automatic detection from X-ray images utilizing transfer learning with convolutional neural networks. Phys. Eng. Sci. Med..

[100] Rahimzadeh M, Attar A, Sakhaei SM (2021). A fully automated deep learning-based network for detecting COVID-19 from a new and large lung CT scan dataset. Biomed. Signal Process. Control.

[101] Deb SD, Jha RK, Jha K, Tripathi PS (2022). A multi model ensemble based deep convolution neural network structure for detection of COVID19. Biomed. Signal Process. Control.

[102] Zhang T, Li X, Qu Z (2022). Lesion attentive thoracic disease diagnosis with large decision margin loss. Biomed. Signal Process. Control.

[103] Çallı E, Sogancioglu E, van Ginneken B, van Leeuwen KG, Murphy K (2021). Deep learning for chest X-ray analysis: A survey. Med. Image Anal..

[104] Barshooi AH, Amirkhani A (2022). A novel data augmentation based on Gabor filter and convolutional deep learning for improving the classification of COVID-19 chest X-Ray images. Biomed. Signal Process. Control.

[105] Koo K-M, Cha E-Y (2017). Image recognition performance enhancements using image normalization. Human-Centric Comput. Inf. Sci..

[106] Johnson AEW (2019). MIMIC-CXR, a de-identified publicly available database of chest radiographs with free-text reports. Sci. Data.

[107] Karargyris A (2021). Creation and validation of a chest X-ray dataset with eye-tracking and report dictation for AI development. Sci. Data.

[108] Nguyen HQ (2022). VinDr-CXR: An open dataset of chest X-rays with radiologist’s annotations. Sci. Data.

[109] Hou J, Gao T (2021). Explainable DCNN based chest X-ray image analysis and classification for COVID-19 pneumonia detection. Sci. Rep..

[110] Danilov VV (2022). Automatic scoring of COVID-19 severity in X-ray imaging based on a novel deep learning workflow. Sci. Rep..

[111] Baltruschat IM, Nickisch H, Grass M, Knopp T, Saalbach A (2019). Comparison of deep learning approaches for multi-label chest X-ray classification. Sci. Rep..

[112] Sadre R, Sundaram B, Majumdar S, Ushizima D (2021). Validating deep learning inference during chest X-ray classification for COVID-19 screening. Sci. Rep..

[113] Munadi K, Muchtar K, Maulina N, Pradhan B (2020). Image enhancement for tuberculosis detection using deep learning. IEEE Access.

[114] Jaeger S (2014). Two public chest X-ray datasets for computer-aided screening of pulmonary diseases. Quant. Imaging Med. Surg..

[115] Msonda P, Uymaz SA, Karaağaç SS (2020). Spatial pyramid pooling in deep convolutional networks for automatic tuberculosis diagnosis. Trait. du Signal.

[116] He, K., Zhang, X., Ren, S. & Sun, J. Deep Residual Learning for Image Recognition. In *2016 IEEE Conference on Computer Vision and Pattern Recognition (CVPR) - 27–30 June 2016,* 770–778 (IEEE, 2016). 10.1109/CVPR.2016.90.

[117] Szegedy, C. *et al.* Going deeper with convolutions. In *2015 IEEE Conference on Computer Vision and Pattern Recognition (CVPR)* 1–9 (IEEE, 2015). 10.1109/CVPR.2015.7298594.

[118] Krizhevsky A, Sutskever I, Hinton GE (2017). ImageNet classification with deep convolutional neural networks. Commun. ACM.

[119] Balas VE, Son LH, Jha S, Khari M, Kumar R (2020). Front matter. Internet of Things in Biomedical Engineering.

[120] Park SH, Goo JM, Jo C-H (2004). Receiver operating characteristic (ROC) curve: Practical review for radiologists. Korean J. Radiol..

[121] Tartaglione E, Barbano CA, Berzovini C, Calandri M, Grangetto M (2020). Unveiling COVID-19 from chest x-ray with deep learning: A hurdles race with small data. Int. J. Environ. Res. Public Health.

[122] Oh Y, Park S, Ye JC (2020). Deep learning COVID-19 features on CXR using limited training data sets. IEEE Trans. Med. Imaging.

