# Supplementary material for: Effective processing pipeline PACE 2.0 for enhancing chest x-ray contrast and diagnostic interpretability
Source: Sci Rep. 2023 Dec 18;13:22471. doi: 10.1038/s41598-023-49534-y (PMC10728198; doi:10.1038/s41598-023-49534-y)
Supplement: Supplementary file 1 — Supplementary Information. [file 41598_2023_49534_MOESM1_ESM.pdf]

Supplementary Material Information File

Effective processing pipeline PACE 2.0 for enhancing chest x-ray contrast and diagnostic interpretability

Giulio Siracusano[1,*], Aurelio La Corte[1], Annamaria Giuseppina Nucera[2], Michele Gaeta[3], Massimo Chiappini[4,5,*] and Giovanni Finocchio[4,6,*]

[1] Department of Electric, Electronic and Computer Engineering, University of Catania, Viale Andrea Doria 6, 95125 Catania, Italy; giuliosiracusano@gmail.com (G.S.); aurelio.lacorte@unict.it (A.L.C.)

[2] Unit of Radiology, Department of Advanced Diagnostic-Therapeutic Technologies, "Bianchi-Melacrino-Morelli" Hospital, Reggio Calabria, Via Giuseppe Melacrino, 21, 89124 - Reggio Calabria, Italy

[3] Department of Biomedical Sciences, Dental and of Morphological and Functional Images, University of Messina, Via Consolare Valeria 1, 98125 Messina, Italy

[4] Istituto Nazionale di Geofisica e Vulcanologia (INGV), Via di Vigna Murata 605, I-00143 Roma, Italy

[5] Maris Scarl, via Vigna Murata 606, 00143 Roma, Italy

[6] Department of Mathematical and Computer Sciences, Physical Sciences and Earth Sciences, V.le F. Stagno D'Alcontres 31, University of Messina, 98166 Messina, Italy

* Corresponding authors: giuliosiracusano@gmail.com; massimo.chiappini@ingv.it; gfinocchio@unime.it

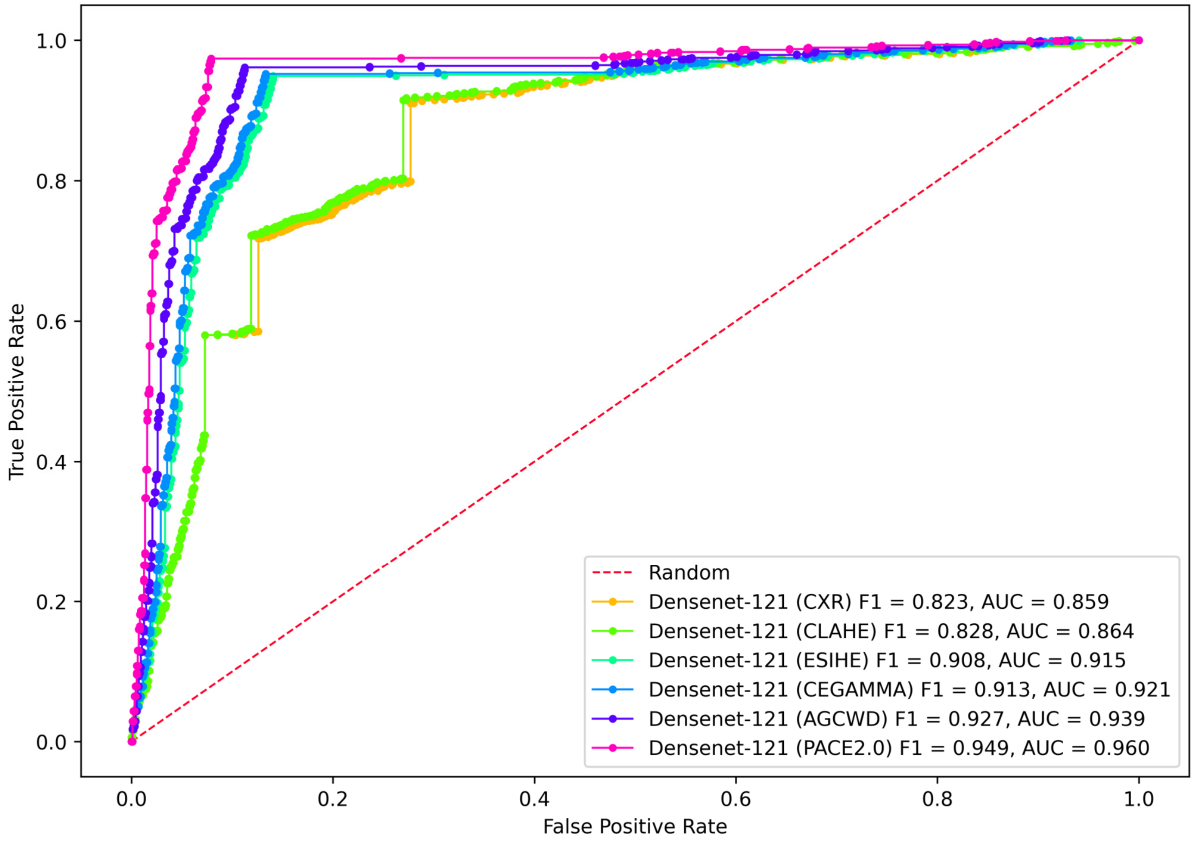

FIG. A1 - Plot of the Receiver Operating Curve (ROC) for a classifier based on transfer learning with a DenseNet-121 model, considering different input configurations: original CXR images (yellow line), CLAHE-processed images (light green line), ESIHE-processed images (green line), CEGAMMA-processed images (blue line), AGCWD-processed images (purple line), and PACE2.0-processed images (violet line). The ROC curve is a powerful tool to analyze the tradeoff between the true positive rate and the false positive rate across a full range of possible thresholds. Comparing the AUC (Area Under Curve) values of the ROC, unprocessed CXRs and CLAHE-processed images exhibit the lowest values at 0.859 and 0.864, respectively. Conversely, AGCWD and PACE2.0 achieve the highest prediction accuracy, with AUC values of 0.939 and 0.960, respectively. These results emphasize the impact of image processing techniques on the classifier's performance and highlight the effectiveness of AGCWD and PACE2.0 in enhancing the model's accuracy.

Presented here is a comprehensive analysis of the Receiver Operating Curve (ROC) for a classifier utilizing transfer learning on a DenseNet-121 model. The ROC plot showcases the performance of the classifier across various input configurations, including original CXR images (represented by the yellow line), as well as images processed using different techniques: CLAHE (light green line), ESIHE (green line), CEGAMMA (blue line), AGCWD (purple line), and PACE2.0 (violet line).
The ROC curve serves as a powerful tool to assess the tradeoff between the true positive rate and the false positive rate across the entire range of possible thresholds.
Upon examining the results, it is evident that the choice of image processing technique significantly impacts the classifier's performance. Unprocessed CXRs (yellow line) and CLAHE-processed images demonstrate relatively lower values for the Area Under the Curve (AUC) of the ROC, scoring 0.859 and 0.864, respectively. Conversely, AGCWD (purple line) and PACE2.0 achieve notably higher

values for prediction accuracy, with AUC scores of 0.939 and 0.960, respectively, indicating their potential as effective approaches for enhancing the classifier's performance.
